# A Practical Risk Score for Prediction of Early Readmission after a First Episode of Acute Heart Failure with Preserved Ejection Fraction

**DOI:** 10.3390/diagnostics11020198

**Published:** 2021-01-29

**Authors:** Marilena-Brîndușa Zamfirescu, Liviu Nicolae Ghilencea, Mihaela-Roxana Popescu, Gabriel Cristian Bejan, Ileana Maria Ghiordanescu, Andreea-Catarina Popescu, Saul G. Myerson, Maria Dorobanțu

**Affiliations:** 1 Cardiothoracic Pathology Department, Carol Davila University of Medicine and Pharmacy, 020021 Bucharest, Romania; brindusa.zamfirescu@gmail.com (M.-B.Z.); crrsty1@yahoo.com (G.C.B.); ileana.ghiordanescu@gmail.com (I.M.G.); andreea.popescu@umfcd.ro (A.-C.P.); maria.dorobantu@gmail.com (M.D.); 2 Department of Cardiology, Elias Emergency University Hospital, “Carol Davila” University of Medicine and Pharmacy, 011227 Bucharest, Romania; 3 Department of Allergology, Elias Emergency University Hospital, 011416 Bucharest, Romania; 4 Oxford Heart Centre, John Radcliffe Hospital, Oxford OX4 2PG, UK; saul.myerson@cardiov.ox.ac.uk; 5 Radcliffe Department of Medicine, University of Oxford, Oxford OX1 2JD, UK; 6 Department of Cardiology, Clinic Emergency Hospital, 011227 Bucharest, Romania

**Keywords:** risk stratification, left ventricle end-diastolic diameter, E/e’ ratio, left ventricle outflow tract velocity-time integral, hospitalization predictor, short-term prognosis, heart failure readmission

## Abstract

Background: The first admission for acute heart failure with preserved ejection fraction (HFpEF) drastically influences the short-term prognosis. Baseline characteristics may predict repeat hospitalization or death in these patients. Methods: A 103 patient-cohort, admitted for the first acute HFpEF episode, was monitored for six months. Baseline characteristics were recorded and their relation to the primary outcome of heart failure readmission (HFR) and secondary outcome of all-cause mortality was assessed. Results: We identified six independent determinants for HFR: estimated glomerular filtration rate (eGFR) (*p* = 0.07), hemoglobin (*p* = 0.04), left ventricle end-diastolic diameter (LVEDD) (*p* = 0.07), E/e’ ratio (*p* = 0.004), left ventricle outflow tract velocity-time integral (LVOT VTI) (*p* = 0.045), and diabetes mellitus (*p* = 0.06). Three of the variables were used to generate a risk score for HFR: LVE**D**D, **E**/e’, LVOT VT**I** -**DEI** Score = **−** 28.763 **+** 4.558 × log (LVE**D**D (mm)) **+** 1.961 × log (**E/**e’ ratio) **+** 1.759 × log (LVOT VT**I** (cm)). Our model predicts a relative amount of 20.50% of HFR during the first 6 months after the first acute hospitalization within the general population with HFpEF with a DEI Score over −0.747. Conclusions: We have identified three echocardiographic parameters (LVEDD, E/e’, and LVOT VTI) that predict HFR following an initial acute HFpEF hospitalization. The prognostic DEI score demonstrated good accuracy.

## 1. Introduction

As the population ages, the number of heart failure (HF) patients appears to be increasing [1,2,3]. Heart failure with preserved ejection fraction (HFpEF) is becoming a common occurrence in daily practice. From the total number of HF patients, around half have HFpEF [4,5], accounting for a considerable burden on the healthcare system, both as in- and outpatients [3]. Over 90% of the patients with HFpEF are ≥60 years old [6] at the time of diagnosis, and as life expectancy increases the public-health impact of HFpEF is likely to follow the same escalating trend.

HFpEF patients do not respond well to the standard treatment used for patients with heart failure with reduced ejection fraction (HFrEF) and have similar mortality rates [7,8,9]. Readmission rates increase in parallel with the average number of days spent in the hospital during the initial hospitalization for acute HFpEF [10]. The complex pathophysiology of HFpEF, the heterogeneity of the patient population, and a large number of comorbidities at the age of onset could explain the limited number of therapeutic options and the poor response to treatment. Both US and European HF guidelines have highlighted the importance of recognizing and managing multiple comorbidities, adjusting treatment to the patient phenotype [4,11]. Furthermore, there is an urgent need to identify predictors and trends of HFpEF readmission, as an initial step towards the personalized management of this specific group of patients.

Echocardiography is invaluable in the risk stratification of patients with HFpEF. It is, however, unclear how clinical and echocardiographic data should integrate into the monitoring and prognostic assessment of HFpEF. This study aimed to identify clinical and echocardiographic predictors of disease progression in HFpEF, focusing on the risk of rehospitalization or death after an index hospital admission. As the first hospital admission for HFpEF has a significant impact on short-term prognosis [12,13], we focused our research on patients in this particular group.

## 2. Materials and Methods

### 2.1. Study Design

This prospective observational study was performed between April 2017 and March 2020 at Elias Emergency University Hospital (EEUH). A total of 103 consecutive patients during their first hospitalization for acute HFpEF and their characteristics have been analyzed. The study protocol complied with the Declaration of Helsinki and was approved by the Ethics Committee of EEUH. All patients provided written informed consent.

The documented data included: cardiovascular risk factors, associated conditions, medication upon discharge, hemoglobin level, estimated glomerular filtration rate (eGFR), blood sodium, and *n*-terminal pro-B type natriuretic peptide (NT proBNP) levels. The echocardiography parameters were assessed within the first 24 h according to the ESC guidelines [14,15,16] using a Vivid T8 Pro (GE Healthcare). Follow-up data were collected at six months.

Patients inclusion criteria were: (1) first hospitalization for acute HF (with clinical signs and symptoms of HF, according to the Framingham criteria) [17], (2) left ventricular ejection fraction (LVEF) ≥50% (assessed by echocardiography with the modified Simpson’s rule) [4], (3) NT-proBNP >220 pg/mL (in sinus rhythm) and >660 pg/mL (in atrial fibrillation) [18,19,20], and at least one additional criterion: (A) left ventricle mass index (LVMI) ≥115 g/m² for males and ≥95 g/m² for females, or (B) diastolic dysfunction (defined as at least 3 of the following: average E/e’>14, septal e’ velocity <7 cm/s or lateral e’ velocity <10 cm/s, tricuspid regurgitation velocity >2.8 m/s, left atrial volume index (LAVI) >34 mL/m²).

Patients exclusion criteria were: (1) significant left heart valve disease (mitral or aortic regurgitation above moderate, mitral or aortic stenosis above mild), (2) severe mitral annulus calcification, (3) acute coronary syndrome, (4) acute pulmonary embolism, (5) pericardial constriction, (6) severe kidney failure (clearance <15 mL/min/1.73 m² or dialysis).

Patient follow-up was performed prospectively at six months after admission. Vital status was assessed through scheduled outpatient department appointments, by phone call, or alternatively during readmissions to our hospital.

### 2.2. End-Points and Study Aim

The primary end-point was the number of heart failure readmissions (HFR). The secondary endpoint was all-cause mortality.

We aimed to identify clinical, biological, and echocardiographic predictors for HFR, and design a prediction score for HFR and all-cause mortality at six months after discharge of patients with first acute event attributable to HFpEF.

### 2.3. Statistical Analysis

Data for continuous variables are presented as mean ± SD (standard deviation) (%) for uniform distribution or as medians (interquartile range (IQR)) for non-uniform distribution. A t-test or Mann-Whitney U rank-sum/ Wilcoxon rank-difference test was used to compare numerical variables between groups.

Categorical data are reported as numbers (percentages %), and group comparisons have been performed with Pearson’s chi-square test and Fischer’s exact test.

The variables that were statistically different between patients with HFR and patients without HFR in the cohort were modeled in a univariate fashion using binary logistic regression in order to identify univariable independent predictors among variables, each at a time, with a *p*-value < 0.05. For each variable we used AUROC (area under the ROC curve) >0.60 and a Hosmer-Lemeshow goodness-of-fit test, *p*-value > 0.05 as criteria to identify independent variables for the model. The validated independent variables were initially transformed using natural logarithm (ln), and were afterward assembled in multivariate models, which were compared for both discrimination and calibration [21]. The estimation of the multivariate model consisted of a backward stepwise approach (*p* < 0.10 to enter, *p* > 0.15 to be removed). The calibration of the predicted models used Akaike’s Information Criterion (AIC) and Bayesian Information Criterion (BIC) as tests for Goodness-of-Fit at the lowest values [21]. The probability of HFR at six months according to the modeled score was computed as a function with the remaining variables included. The validation of the results was performed after a random selection of an internal validation contingent of 46 patients from the studied cohort [21].

An optimal threshold was identified in the training cohort (with a maximum Youden index), and the sensitivity and specificity identified were reported. The difference between the two AUROC curves of the training (study) cohort and the validation contingent was calculated using the Hanley & McNeal test.

The odds ratio (OR) was generated for each of the variables identified.

The Kaplan-Meier method was applied to create survival estimates. A Chi-square test was also used to compare the rates of HFR and death between the two groups. All *p*-values were two-sided and a *p*-value < 0.05 was considered statistically significant. The statistical analysis was performed with SPSS version 26 (Statistical Package for Social Science, IBM, Armonk, NY, USA: IBM Corp.).

## 3. Results

### 3.1. Study Cohort

**Figure 1:**
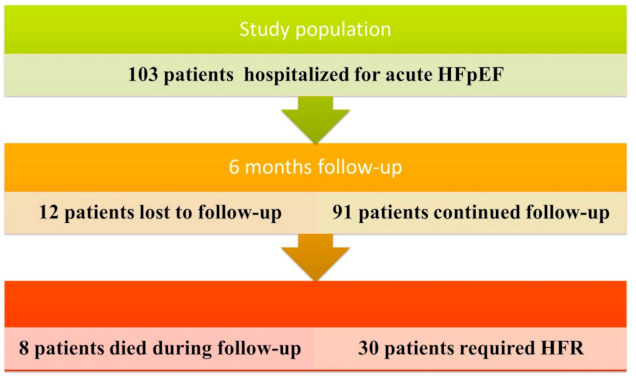
Patient flow-chart at six months. HFR: heart failure readmission, HFpEF: Heart failure with preserved ejection fraction.

The study population included 103 hospitalized patients in EEUH between April 2017 and October 2019. Within six months twelve patients were lost to follow-up and eight patients died. Thirty patients were readmitted due to acute decompensation of HFpEF (See Figure 1).

### 3.2. Baseline Characteristics

Overall, 62 women (68%), and 29 men with a mean age of 73.0 years (±10 years) have been included in the study. Demographic, clinical, and laboratory key baseline characteristics of the patients with and without HFR are summarized in Table 1.

A significant proportion of the study population had comorbidities including known cardiovascular risk factors such as: obesity (63%), arterial hypertension (100%), coronary artery disease (23%), hypercholesterolemia (82%), diabetes mellitus (56%), paroxysmal or persistent atrial fibrillation (69%), and chronic kidney disease (43%). Besides the cardiovascular conditions, the patients’ medical records included other comorbidities, such as chronic obstructive pulmonary disease (13%), asthma (8%), sleep apnoea syndrome (13%), and cerebrovascular disease (19%). Thirty-nine patients (42%) had impaired lung function tests.

The mean/median values for echocardiographic parameters were calculated, see Table 2.

### 3.3. Clinical Features and Outcomes

The clinical presentation of acute HFpEF syndrome was mainly as acute left heart failure (75%) while 25% of the patients presented with predominantly right heart failure. 23% of these patients required respiratory support either as invasive ventilation (6.6%) or non-invasive positive pressure ventilation (22%). The median duration of the index hospitalization was 7.5 (IQR = 5) days.

After six months of follow-up, 30 patients (33%) required HFR, and eight patients (9%) died. Of the 30 patients needing heart failure readmission, two also suffered a stroke. Mortality was classified as: cardiovascular (63%), non-cardiovascular (25%) and of unknown cause (12%).

### 3.4. Independent Predictors for Short Term HFR

We identified six independent determinants for HFR at six months with a difference (*p* < 0.10) between the two groups of the cohort (with and without HFR at six months). The determinants are: E/e’ ratio, level of hemoglobin, left ventricular outflow tract velocity time integral (LVOT VTI), LV end-diastolic diameter (LVEDD), eGFR, and presence of DM. These variables had the highest value at Hosmer and Lemeshow test and an AUC over 0.600 (see Table 3, Table A1). However, these predictors for HFR at six months did not seem to have an influence on all-cause mortality (See Table 1).

**Figure A1:**
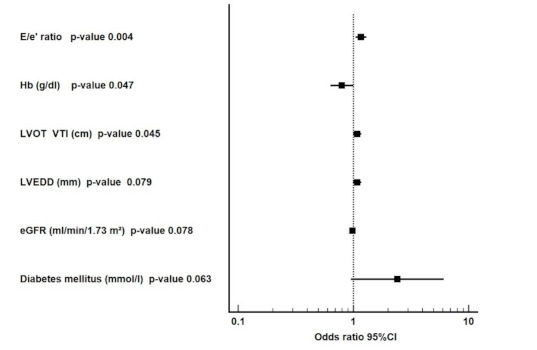
Forest plot for HFR predictors at 6 months. Point estimates represent the multivariable odds ratio for HFR after six months; whiskers indicate a 95% confidence interval (CI).

Next, the odds ratio (OR) of the six selected clinical and echocardiographic characteristics for HFR at six months were calculated with univariate analysis and are detailed in Table A3, Figure A1.

### 3.5. Modeling the Score

The six aforementioned parameters, with statistically significant OR for causing an early HFR, were included and computed in a binary logistic regression, with a backward approach in order to identify the predictors to be included in a risk score for readmission at six months after the first hospitalization for acute HFpEF.

The power of prediction of each variable, considered for the role of predictor in the score, was assessed according to the coefficient of determination (Nagelkerke R-square value) between the outcome and each variable determinant.

The model was constructed with binary regression with a backward stepwise method, which started with a model that included all the six independent predictors. At each step a predictor was eliminated from the model, using the Nagelkerke R square and Hosmer & Lemeshow tests for the whole model [21].

In the first step, the initial model (model 1) incorporated all the six variables considered to be predictors: E/e’ ratio, level of hemoglobin, LVOT VTI, LVEDD, eGFR, and presence of DM. In the second step, eGFR was eliminated; the second model loses a statistically insignificant (*p* = 0.907) power of prediction (−0.014) compared to model 1 (with all six predictors). In the third step, DM was rejected, as model three has lost −0.707 of its prediction power, with no statistical significance (*p* = 0.401). In step four, Hb was removed from the general model, with a loss of −1.220 of the power of prediction (*p* = 0.269). The general observation was that three variables were cast away from the general model with no significant loss of prediction power, which proves that eGFR, DM, and Hb are not predictors of the model. The other three variables that remained (LVEDD, E/e’ ratio, LVOT VTI) were taken into consideration for the final model of predicting the HFR at six months (see Table A2).

**Figure 2:**
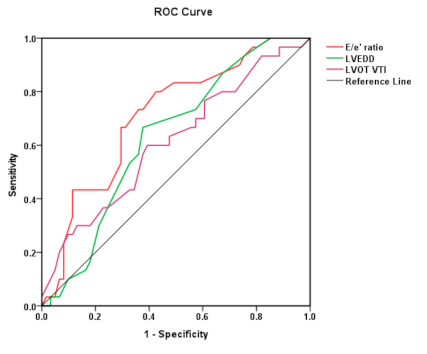
The ROC curve for each of the three independent determinants (LVEDD—green, E/e’ ratio—red, LVOT VTI—purple) for HFR at 6 months. AUC are as follows: E/E’ ratio = 0.710 (*p* = 0.001), LVEDD = 0.630 (*p* = 0.045), LVOTVTI = 0.611 (*p* = 0.085).

All the three mentioned predictors have an AUC over 0.600 (0.630; 0.710; 0.611), with good statistical significance (*p* = 0.059; 0.056; 0.063) for LVEDD, E/e’ ratio, and LVOT VTI respectively (see Figure 2).

**Figure 3:**
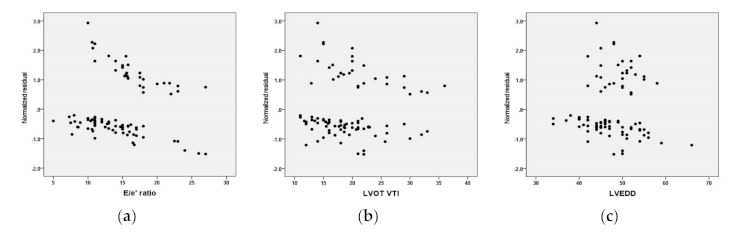
The normalized residuals for the model’s predictors: (**a**) E/e’ ratio, (**b**) LVOT VTI—left ventricular outflow tract velocity time integral, (**c**) LVEDD- left ventricle end-diastolic diameter.

The validation of each predictor variable was assessed by computing the normalized residuals for each of them (see Figure 3). There is no relationship between each of the variables assumed to be predictors of our model and the residuals of the final model, which means each variable forecasts correctly the HFR at six months.

The next step in modeling the score was to assess the out-of-the-sample prediction error and thereby the relative quality of statistical models for our set of data using the AIC (Aikake’s Information Criterion) and BIC (Bayesian Information Criterion) (see Table A4). We have selected model 4 with three predicting variables: E/e’ ratio, EDDLV, and LVOT VTI, of all the four prediction models presented in Table A4, according to the lowest AIC and BIC values for the model compared with the three previous models.

### 3.6. The DEI Score Model

The acronym for the modeled score is DEI, and was constructed based on the components of the scoring system: D (left ventricle end-**D**iastolic diameter), E (**E**/e’ratio), I (left ventricle outflow tract velocity-time **I**ntegral).

The variables used to build the model must fulfill strict criteria at univariate regression such as AUROC > 0.60 and a Hosmer–Lemeshow *p*-Value > 0.05. Non-parametric variables were transformed into parametric variables using natural logarithmic functions. Overall the model proved to be much more balanced and with a better predictive power than each other of the univariate models (see Table 4 and Table A4).

The computation of the DEI score is made according to the following formula:

DEI Score = − 28.763 + 4.558 × log (LVEDD (mm)) + 1.961 × log (E/e’ ratio) + 1.759 × log (LVOT VTI (cm))

The cut-off of the DEI score for HFR at six months is over −0.747 (Sensitivity = 73.33%, 95% CI = 54.10–87.70, Specificity = 72.13%, 95% CI = 59.20–82.90) with a positive LR = 2.63 and a negative LR of 0.37 (35).

**Figure 4:**
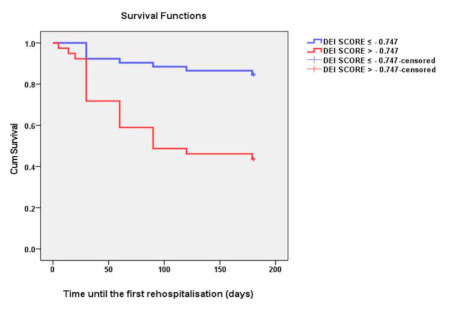
Kaplan-Meier curves according to DEI score for HFR (time in days to readmission) at six months in patients with acute hospitalized HFpEF. The two Kaplan-Meier curves are significantly apart (*p* = 0.001). Low DEI score (≤−0.747) in blue, high DEI score (>−0.747) in red.

The Kaplan-Meier curve for HFR according to the DEI score showed that patients with a score over −0.747 presented a statistically significantly higher number of HFR at six months compared to those with a score below −0.747 (Log Rank test *p* = 0.001) (see Figure 4).

A relative amount of 20.50% of HFR during the first six months of follow-up is predicted by our model within the general population with acute HFpEF and a DEI score over −0.747.

### 3.7. Validation of the DEI Score

**Figure 5:**
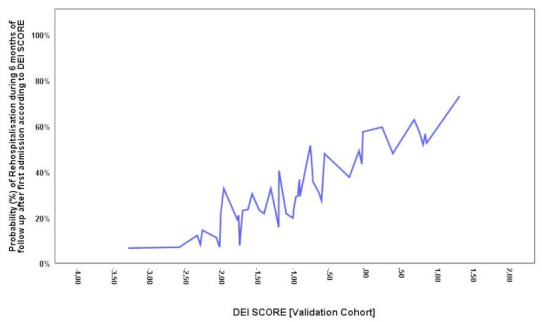
Probability plot of the rehospitalization during six months of follow-up after the first admission according to DEI score, according to the validation cohort.

We applied the DEI Score to the validation contingent initially randomized (www.random.com) (See Figure 5). The validation cohort comprised 46 patients, with a ratio of HFR and non-HFR similar to that of the training cohort (1:2).

**Figure 6:**
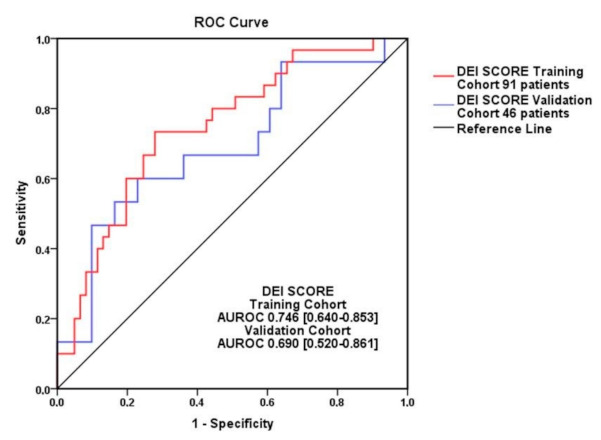
ROC curves of the training cohort (91 patients-red) and validation cohort (46 patients-blue). The two AUROC show close values and no significant differences (Hanley & McNeil test, *p* = 0.47).

**Figure A2:**
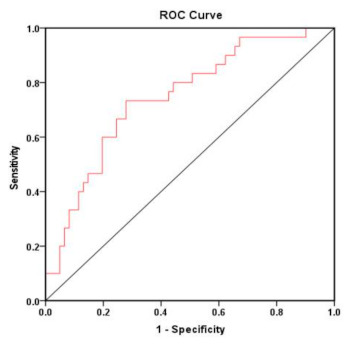
The ROC curve for the values of the DEI Score in the training cohort according to HFR during the 6 months from the first episode of HFpEF. AUROC = 0.746, 95% CI (0.640–0.853), *p* = 0.0001.

**Figure A3:**
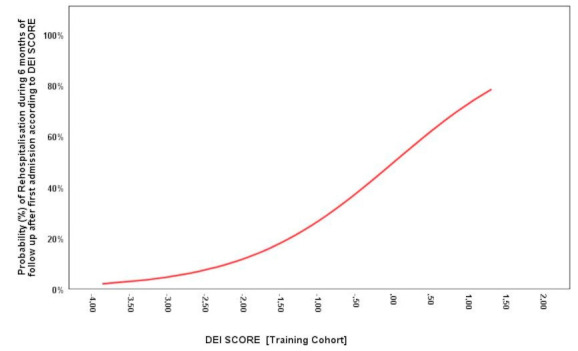
Probability plot of the HFR during 6 months of follow up after the first admission according to the DEI score.

The validation of the model was assessed further by using ROC curves of the training cohort (AUROC = 0.746, 95% CI = 0.640–0.853, *p* = 0.0001) (see Figure A2 and Figure A3) and validation group (AUROC = 0.690 (95% CI = 0.520–0.861, *p* = 0.038). We computed the possibility of predicting the HFR at six months by comparing AUROC of the training cohort and validation cohort (see Figure 6). The Hanley Mc Neil test has a *p*-value = 0.47, which means that there is no statistical difference between the two ROC curves of the DEI score (the training cohort of 91 patients and the 46 patients validation contingent) in regard to the HFR at six months after a first acute event of HFpEF.

We performed an analysis of the DEI Score in the training cohort based on its cut-off values in terms of maximizing sensitivity, specificity, or according to the Youden’s Index criteria, and the result is reported in Table A5: (A) a DEI Score ≤ −2.605 showed a 100% sensitivity, 9.84% specificity, 5.5% PPV, 100% NVP, 1.11 +LR; (B) a DEI Score ≥ −0.747 showed a 73.33% sensitivity, 72.13% specificity, 12.2% PPV, 98.1% NVP, 2.63 +LR, 0.37 −LR; (C) a DEI Score ≥ 0.812 showed a 10% sensitivity, 100% specificity, 100% PPV, 95.6% NVP, 0.90 −LR.

We applied this pattern of validation according to cut-off values in the analysis of DEI Score to the validation cohort and noticed a comparable performance.

## 4. Discussion

**Rationale for the study.** As the world’s population continues to age, a thorough understanding of the characteristics and outcomes of patients with acute HFpEF becomes crucial in reducing the burden of morbidity and mortality caused by this affliction. Therefore the development of risk prediction tools can prove effective in guiding the medical decision for these patients. Consequently, patients estimated to be at higher risk for rehospitalization or death may be treated in a personalized manner.

**Added value to current literature.** To date, there is a limited number of studies that specifically address heart failure with preserved ejection fraction, especially since the current registries report on heart failure, in general, as one entity [22,23]. The current literature also largely fails to concentrate on the subject of HFpEF with acute presentations.

To the best of our knowledge, the current study is the first to include only patients hospitalized for the first time with acute HFpEF. The study looks for prognostic and prediction tools to be used in this group of patients, considering that the specific moment of the first hospital admission is a turning point in the evolution of heart failure.

The DEI risk score, based on echocardiographic predictors, could prove to be a convenient instrument to evaluate the patient’s prognosis and, at the same time, prescribe recommendations for follow-up as early as the initial presentation in the emergency department. These features render it a useful tool, with good accuracy for the management of patients suffering from acute heart failure with preserved ejection fraction.

In the era of COVID-19, when emergency rooms and intensive care facilities are facing high pressure, we propose assessment of patients with acute HFpEF by means of the DEI risk score in order to estimate their short-term prognosis and those in need of closer monitoring.

**Comparison to similar studies.** The study population and several characteristics were found to be relatively similar to other studies. For instance, lower hemoglobin levels seemed to enhance the need for rehospitalization in our study group, as previously demonstrated [24]. Moreover, lower hemoglobin levels have been associated with a longer hospital stay. Studies using machine learning have managed to identify prediction models for chronic HFpEF [25] that included the hemoglobin level and the glomerular filtration rate, two parameters we have also found to be of importance in our population.

Our study identified some interesting correlations between echo-graphic findings and the likelihood of rehospitalization at six months for patients with acute HFpEF. Our finding that LVOT VTI is a determinant of HFR, with higher values predicting HFR, is in contrast with a recent study of Omote et al. which associated a lower value with a worse outcome [26]. However, Omote et.al used a composite index (all-cause mortality and HFR) and followed the patients in the long-term. The present study focused on the first six months, and the outcome was HFR alone, not a composite index. Our data show that early on in the evolution of HFpEF, a slightly higher LVOT VTI is associated with more hospitalizations. This might indicate that, in the course of the disease, LVOT VTI does not show a linear decline. After an initial adaptative rise, linked to an increased left ventricular mass and increased filling pressures associated with more hospitalizations, the LVOT VTI starts to decline and the prognosis is worse in the long term. The higher ventricular mass is explained in part by the fact that 100% of our study population was hypertensive, thus explaining a worse relaxation profile.

Currently, risk assessment is based on a combination of clinical biomarkers and several generally recognized echocardiographic parameters [27]. Still, risk prediction can be improved by including left ventricle echocardiographic parameters such as LVEDD, LVOT VTI, and the E/e’ ratio in the diagnostic algorithm. The applicability of non-sophisticated, accessible parameters such as these is what renders the DEI score a useful clinical instrument.

Ultimately, in our study, the predictors for HFR did not correlate with all-cause mortality which was not an unexpected finding since other studies have demonstrated that the predictors for rehospitalization and mortality are not the same [28]. On the other hand, a six months follow-up may not be enough to allow mortality predictors identification.

**Study limitations**. The calculations were made on a limited number of patients from a single-center. The relatively small number of patients was due to the strict inclusion criteria. Moreover, we included only patients hospitalized for the first episode of acute HFpEF. The follow-up period is short because the focus of our study was early heart failure readmissions. However, the monitoring will continue for another 18 months. A potential limit of the present study is the lack of evaluation of pulmonary congestion using lung ultrasound (LUS) that was shown to be of prognostic value in recent studies [29,30].

**Future directions**. The population characteristics, their correlations, and the risk score identified in this study need to be further addressed in larger randomized studies of acute HFpEF. Furthermore, a comparison between outpatients with HFpEF and patients presenting to the hospital with acute HFpEF would bring additional important information. We also plan to instate an online calculator for the DEI score.

## 5. Conclusions

Early rehospitalization of patients with a first acute HFpEF event is predicted by several echocardiographic parameters included in the DEI score ((left ventricle end-**D**iastolic diameter), (**E**/e’ratio), (left ventricle outflow tract velocity-time **I**ntegral).). However, all-cause mortality does not seem to be influenced by these heart failure readmission predictors in the short-term.

## Figures and Tables

**Table 1:** Complete baseline characteristics of the patients with acute HfpEF according to HFR status (*n* = 91).

Characteristics	No HFR	HFR	*p*-Value
Number (%)	61 (67.03)	30 (32.96)	
Age at diagnosis, yo, mean ± SD (95% CI)	73.57 ± 10.85 (70.80–75.00)	71.97 ± 10.21 (68.16–75.78)	0.50 *
Female gender, *n* (%)	39 (63.90%)	23 (76.70%)	
Male gender, *n* (%)	22 (36.10%)	7 (23.30%)	
Cardiovascular risk factors			
High blood pressure, *n* (%)	61 (100%)	30 (100%)	1
Diabetes mellitus, *n* (%)	30 (49.2%)	21 (70%)	0.06 **
Tobacco smoking (current or former), *n* (%)	17 (27.90%)	8 (26.70%)	0.90 **
Hypercholesterolemia, *n* (%)	48 (78.70%)	27 (90%)	0.183 **
BMI, median (IQR)	31 (9)	31.50 (7)	0.351 *
Previous medical history			
Medical history of CAD, *n* (%)	14 (23%)	7 (23.30%)	0.968 **
Medical history of MI, *n* (%)	9 (14.80%)	4 (13.30%)	0.856 **
Medical history of stroke, *n* (%)	10 (16.40%)	8 (26.70%)	0.247 **
History of Atrial fibrillation, *n* (%)	44 (72.10%)	19 (63.30%)	0.393 **
Medical history of lung disease, *n* (%)	27 (44.30%)	17 (56.70%)	0.266 **
Medical history of sleep apnea, *n* (%)	6 (9.80%)	6 (20%)	0.178 **
Assessment on admission			
Non-Invasive ventilation on admission, *n* (%)	12 (19.70%)	8 (26.70%)	0.449 **
Mechanical ventilation on admission, *n* (%)	5 (8.20%)	1 (3.30%)	0.379 **
Peripheral edema on admission, *n* (%)	32 (52.50%)	21 (70%)	0.111 **
SaO_2_ on admission, median (IQR)	90 (7)	88.50 (5)	0.378 ***
HR on admission, median (IQR)	96 (53)	101 (51)	0.609 ***
SBP, mm Hg, mean ± SD (95% CI)	185.25 ± 33.83 (176.58–193.91)	185.83 ± 37.18 (171.95–199.72)	0.94 *
DBP, mm Hg, mean ± SD (95% CI)	98.10 ± 17.18 (93.70–102.50)	101.50 ± 19.83 (94.09–108.91)	0.40 *
Serum natremia, mmol/L, median (IQR)	140 (6)	139 (3)	0.141 ***
eGFR, mL/min/1.73 sqm, mean ± SD (95% CI)	70.29 ± 28.64 (62.95–77.62)	58.86 ± 27.92 (48.43–69.29)	0.075 *
Hb, g/dl, mean±SD (95% CI)	12.27 ± 2.12 (11.73–12.91)	11.36 ± 1.70 (10.72–11.99)	0.043 *
NTproBNP, ng/L, median (IQR)	3563 (6907)	2928 (4256)	0.886 ***
Length of in-hospital stay, days, median (IQR)	7 (5)	8.50 (4)	0.135
Mortality of any cause at six months, *n* (%)	5 (8.20%)	3 (10%)	0.775

Data are presented as mean ± SD (%), medians, and as numbers (percentages). 95% CI = 95% confidence interval of the difference; IQR: interquartile range. * The *p*-value was calculated with *t*-test; ** The *p*-value was assessed using Pearson Chi Square test for non-parametric variables such as percentages of occurrence of an ordinal or nominal variable, *** The *p*-value was assessed using Mann-Whitney U test for the continuous variables with abnormal distribution, where skewness and kurtosis were outside the range (−1, +1) and (−2, +2), respectively; ACEI: angiotensin-converting enzyme inhibitors, ARB: angiotensin receptor blockers, BMI: body mass index, CAD: Coronary Artery Disease; DBP: diastolic blood pressure, eGFR: estimated glomerular filtration rate, Hb: Hemoglobin, HR: heart rate, IQR: interquartile range, MI: Myocardial Infarction, SaO2: arterial oxygen saturation, SBP: systolic blood pressure.

**Table 2:** Transthoracic echocardiography features in patients with HFpEF, with and without HFR.

Characteristics	No HFR	HFR	*p*-Value
Left ventricle			
LV end-diastolic diameter, mm, mean ± SD (95% CI)	46.92 ± 5.97 (45.39–48.45)	49.10 ± 4.02 (47.60–50.60)	0.074 *
LVEF on admission, vol%, mean ± SD (95% CI)	55.62 ± 5.43 (54.23–57.01)	56.27 ± 6.95 (53.67–58.86)	0.63 *
Septal s velocity, cm/s, mean ± SD (95% CI)	6.31 ± 1.23 (5.99–6.62)	6.03 ± 1.11 (5.61–6.45)	0.224 ***
Lateral s velocity, cm/s, mean ± SD (95% CI)	7.37 ± 1.28 (7.04–7.70)	7.28 ± 1.35 (6.77–7.78)	0.918 ***
Left ventricle mass, g/m^2^, median (IQR)	120 (36)	127 (37.25)	0.422 ***
LVOT VTI, cm, mean ± SD (95% CI)	18.60 ± 1.74 (17.29–19.91)	21.19 ± 6.41 (18.80–23.59)	0.04
LA index volume, mL, mean ± SD (95% CI)	50.83 ± 11.86 (47.79–53.87)	53.36 ± 12.61 (48.65–58.06)	0.353 *
E/e’ ratio, median (IQR)	12.8 (5.25)	15.75 (4.50)	0.001 ***
E/e’ > 9, *n* (%)	52 (85.20%)	30 (100%)	0.027 **
E/e’ > 14, *n* (%)	25 (41%)	22 (73.30%)	0.004 **
Septal e’ velocity < 7 cm/s, *n* (%)	42 (68.90%)	23 (76.70%)	0.438**
Lateral e’ velocity < 10 cm/s, *n* (%)	39 (69.60%)	25 (83.30%)	0.168 *
Right ventricle, IVC, and right atrium			
Free wall RV S < 9.5, *n* (%)	8 (13.10%)	4 (13.30%)	0.977 **
TAPSE < 17 mm, *n* (%)	16 (26.20%)	7 (23.30%)	0.765 **
RA area over 18 cm^2^, *n* (%)	44 (72.10%)	19 (63.30%)	0.393 **
IVC over 21 mm, *n* (%)	28 (45.90%)	12 (40%)	0.594 **
IVC collapse < 50%, *n* (%)	25 (41%)	10 (33.30%)	0.481 **
PAPS, mm Hg, mean ± SD (95% CI)	41.22 ± 13.67 (37.72–44.73)	39.90 ± 17.42 (33.39–46.40)	0.692 *
PAPS > 35 mm Hg, *n* (%)	43 (70.50%)	16 (53.30%)	0.107 **

Data are presented as mean ± SD (%), medians, and as numbers (percentages). 95% CI = 95% confidence interval of the difference. IQR: interquartile range. * The *p*-value was calculated with *t*-test; ** The *p*-value was assessed using Pearson Chi-Square test for non-parametric variables such as percentages of occurrence of an ordinal or nominal variable, *** The *p*-value was assessed using Mann-Whitney U test for the continuous variables with abnormal distribution, where skewness and kurtosis were outside the range (−1, +1) and (−2, +2), respectively. HF: heart failure, Hb: Hemoglobin, HR: heart rate, IQR: interquartile range, IVC: inferior vena cava, MI: Myocardial Infarction, PAPS: systolic pulmonary artery pressure, SaO2: arterial oxygen saturation, SBP: systolic blood pressure, TAPSE: tricuspid annular plane systolic excursion, LA: left atrium, LV: left ventricle; LVEF: Left ventricle ejection fraction, LVOT VTI: left ventricular outflow tract velocity time integral; RA: right atrium, RV: right ventricle.

**Table 3:** Selection of the independent variables by bivariate linear regression/univariate analysis.

Variables	Nagelkerke R Square	Hosmer and Lemeshow Test	*p*-Value Regression Coefficient	AUC	*p*-Value AUC
E/e’ ratio	0.136	0.148	0.004	0.710	0.001
Hb	0.062	0.305	0.047	0.665	0.011
LVOT VTI	0.063	0.888	0.045	0.611	0.085
LVEDD	0.049	0.379	0.079	0.630	0.045
eGFR	0.050	0.584	0.078	0.623	0.056
Diabetes mellitus	0.054	N/A	0.063	0.604	0.108
Serum sodium	0.018	0.124	0.284	0.595	0.143
Lateral s velocity	0.002	0.207	0.745	0.507	0.919
Septal s velocity	0.017	0.343	0.296	0.576	0.239
Medium s velocity	0.009	0.652	0.444	0.523	0.717
BMI	0.013	0.806	0.349	0.545	0.483
LV mass	0.006	0.364	0.524	0.552	0.423
NTproBNP	0.006	0.031	0.550	0.695	0.101

AUC: area under the curve; BMI: body mass index; eGFR: estimated glomerular filtration rate; Hb: hemoglobin; LV: left ventricle; LVOT VTI: left ventricular outflow tract velocity time integral; MI: Myocardial infarction; N/A: not applicable.

**Table 4:** Multivariate Model Information- statistical characteristics of model 4.

Multivariate Model	Variable 1 log_e_ (E/e’ Ratio)	Variable 2 log_e_ (LVEDD)	Variable 3 log_e_ (LVOT VTI)
VIF	1.147	1.113	1.139
Coefficient	1.961	4.558	1.759
Coefficient-Standard Error	0.860	2.488	1.002
Coefficient- Significance	*p* = 0.023	*p* = 0.067	*p* = 0.079
Intercept		−28,763	
Intercept-Standard Error		10.951	
Intercept- Significance		*p* = 0.009	
AUROC		0.746 (0.640—0.853)	
Nagelkerke Pseudo-R²		0.228	
Hosmer-Lemeshow *p*-value		0.760	
Akaike’s Information Criterion (AIC)		107.106	
Bayesian Information Criterion (BIC)		117.150	

The regression equation for our model (LVEDD, E/e’ ratio, LVOT VTI) is: Log ODDS RATIO (for HFR at 6 months) = −8.394 + 0.114 × E/e’ + 0.088 × LVEDD + 0.087 × VTI LVOT.

## Data Availability

All data is on hospital records, all data is available on request.

## Appendix A

**Table A1:** Univariate analysis. Intracohort statistically significant variables were studied univariately.

Univariate Analysis	E/e’ Ratio	Hb	LVOT VTI	LVEDD	eGRF	DM
Intercept (β0)	−3.015	2.012	−2.310	−4.345	0.249	−1.237
Intercept (β0)-Standard Error	0.852	1.378	0.840	2.079	0.573	0.379
Intercept (β0)-Significance (*p*-value)	<0.001	0.144	0.006	0.038	0.664	0.210
Coefficient (β1)	0.154	−0.230	0.081	0.076	−0.015	0.880
Coefficient (β1)- Standard Error	0.054	0.116	0.04	0.043	0.008	0.474
Coefficient (β1)- Significance (*p*-value)	0.004	0.047	0.045	0.079	0.078	0.063
AUROC	0.710 (0.600–0.821)	0.665 (0.550–0.780)	0.611 (0.487–0.736)	0.630 (0.514–0.745)	0.623 (0.497–0.750)	0.604 (0.482–0.726)
Nagelkerke Pseudo-R²	0.136	0.062	0.063	0.049	0.050	0.054
Hosmer-Lemeshow *p*-value	0.148	0.305	0.888	0.379	0.584	
AIC	70.532	91.807	68.154	51.665	110.504	12.162
BIC	75.554	96.828	73.176	56.687	115.525	17.184

Hb: hemoglobin; LVOT VTI: left ventricular outflow tract velocity time integral; LVEDD: left ventricular end-diastolic diameter; eGRF: estimated glomerular filtration rate; DM: diabetes mellitus.

**Table A2:** Variables in the Equation of the model, backward approach.

		B	S.E.	Wald	df	Sig.	Exp(B)	95% C.I. for EXP(B)	
								Lower	Upper
Step 1 ^a^	E/e’	0.093	0.058	2.572	1	0.109	1.098	0.980	1.230
	VSTD	0.098	0.053	3.427	1	0.064	1.103	0.994	1.223
	VTI_LVOT	0.058	0.052	1.263	1	0.261	1.060	0.958	1.173
	Hb	−0.130	0.135	0.920	1	0.337	0.878	0.673	1.145
	eGFR	−0.001	0.010	0.014	1	0.907	0.999	0.979	1.019
	DM	−0.465	0.599	0.601	1	0.438	0.628	0.194	2.034
	Constant	−6.162	3.430	3.229	1	0.072	0.002		
Step 2 ^a^	E/e’	0.093	0.058	2.603	1	0.107	1.098	0.980	1.230
	VSTD	0.099	0.052	3.607	1	0.058	1.104	0.997	1.222
	VTI_LVOT	0.059	0.051	1.304	1	0.253	1.060	0.959	1.173
	Hb	−0.135	0.129	1.099	1	0.295	0.874	0.679	1.125
	DM	−0.483	0.578	.699	1	0.403	0.617	0.199	1.915
	Constant	−6242	3.368	3.435	1	0.064	0.002		
Step 3 ^a^	E/e’	0.107	0.056	3.624	1	0.057	1.112	0.997	1.241
	VSTD	0.091	0.050	3.282	1	0.070	1.095	0.993	1.208
	VTI_LVOT	0.070	0.049	1.974	1	0.160	1.072	0.973	1.181
	Hb	−0.141	0.129	1.194	1	0.275	0.869	0.675	1.118
	Constant	−6.390	3.345	3.649	1	0.056	0.002		
Step 4 ^a^	E/e’	0.114	0.055	4.314	1	0.038	0.121	1.006	1.248
	VSTD	0.088	0.050	3.141	1	0.076	1.092	0.991	1.204
	VTI_LVOT	0.087	0.047	3.450	1	0.063	1.091	0.995	1.196
	Constant	−8.394	2.860	8.616	1	0.003	0.000		

^a^ Variable(s) entered on step 1: E/E’, VSTD, VTI_LVOT, Hb, eMDRD, DMtotal.

**Table A3:** Odds ratio (OR) for HFR at six months.

Characteristics	Odds Ratio	95% CI	*p*-Value *
DM present	2.411	0.953–6.101	0.06
Serum sodium	0.949	0.864–1.044	0.284
eGFR (mL/min/1.73 sqm)	0.985	0.969–1.002	0.078
Hb (g/dL)	0.794	0.633–0.997	0.047
LVEDD (mm)	1.079	0.991–1.174)	0.079
E/e’ ratio	1.167	1.050–1.297	0.004
LVOT VTI (cm)	1.084	1.002–1.173	0.045

* *p*-value (Pearson chi-square); 95% CI: 95% confidence interval.CVR: cardiovascular readmission; DM: diabetes mellitus; eGFR: estimated glomerular filtration rate; Hb: hemoglobin; LVEDD: left ventricle end-diastolic diameter; LVOT VTI: left ventricle outflow tract velocity-time integral.

**Table A4:** Goodness of Fit assessment of the models. The AIC and BIC tests.

Nr.	MODEL	AIC	AICC	BIC	Nagelkerke R Square	Hosmer Lemeshow Test	*p*-Value Test OMNIBUS
1	E/e’ Hb VSTD VTI LVOT DM eGFR	112.937	114.286	130.513	0.230	0.108	0.012
2	E/e’ VSTD VTI LVOT Hb DM	110.951	111.951	126.016	0.230	0.033	0.006
3	E/e’ VSTD VTI LVOT Hb	109.657	110.363	122.212	0.221	0.204	0.003
4	E/e’ VSTD VTI LVOT	108.877	109.342	118.920	0.205	0.881	0.002

**Table A5:** Cut-off model. The criteria for choosing values of cut-off points in the training cohort were represented by maximizing either sensitivity or specificity or according to the Youden’s Index criteria.

Cut-Off Value Selection	Maximize Sensitivity ≤ 2.605		Maximize Specificity > 0.812		Youden’s Index = 0.454 Associated Criterion > 0.747	
	Training	Validation	Training	Validation	Training	Validation
Sensitivity (%)	100 (88.4–100)	100 (78.2–100)	10 (2.1–26.5)	13.33 (1.7–40.5)	73.33 (54.1–87.7)	60 (32.3–83.7)
Specificity (%)	9.84 (3.7–20.2)	6.45 (0.8–21.4)	100 (94.1–100)	100 (88.8–100)	72.13 (59.2–82.9)	77.42 (58.9–90.4)
PPV (%)	5.5 (5.1–6)	5.3 (4.9–5.8)%	100	100	12.2 (8.1–18)	12.3 (6.1–23.2)
NPV (%)	100	100	95.5 (94.9–96)	95.6 (94.7–96.4)	98.1 (96.5–99)	97.4 (95.1–98.6)
+LR	1.11 (1–1.1)	1.07 (1–1.2)	*	*	2.63 (1.7–4.2)	2.66 (1.2–5.7)
−LR	0	0	0.90 (0.8–1)	1 (1.0–1.0)	0.37 (0.2–0.7)	0.52 (0.3–1.0)

* very high value; PPV: positive predictive value; NPV: negative predictive value; +LR: positive likelihood ratio; −LR: negative likelihood ratio.

## References

[1] Bleumink G.S., Knetsch M., Sturkenboom M.C.J.M., Straus S.M.J.M., Hofman A., Deckers J.W., Witteman J.C.M., Stricker B.H.C. (2004). Quantifying the heart failure epidemic: Prevalence, incidence rate, lifetime risk and prognosis of heart failure—The Rotterdam Study. Eur. Heart J..

[2] Owan T.E., Hodge D.O., Herges R.M., Jacobsen S.J., Roger V.L., Redfield M.M. (2006). Trends in prevalence and outcome of heart failure with preserved ejection fraction. N. Engl. J. Med..

[3] Hashemi D., Dettmann L., Trippel T.D., Holzendorf V., Petutschnigg J., Wachter R., Hasenfuß G., Pieske B., Zapf A., Edelmann F. (2020). Economic impact of heart failure with preserved ejection fraction: Insights from the ALDO-DHF trial. ESC Heart Fail..

[4] Ponikowski P., Voors A.A., Anker S.D., Bueno H., Cleland J.G.F., Coats A.J.S., Falk V., González-Juanatey J.R., Harjola V.P., Jankowska E.A. (2016). 2016 ESC Guidelines for the diagnosis and treatment of acute and chronic heart failure. Eur. Heart J..

[5] Arora S., Lahewala S., Hassan Virk H.U., Setareh-Shenas S., Patel P., Kumar V., Tripathi B., Shah H., Patel V., Gidwani U. (2017). Etiologies, trends, and predictors of 30-day readmissions in patients with diastolic heart failure. Am. J. Cardiol..

[6] Borlaug B.A. (2014). The pathophysiology of heart failure with preserved ejection fraction. Nat. Rev. Cardiol..

[7] Metra M., Teerlink J.R., Cotter G., Davison B.A., Felker G.M., Filippatos G., Greenberg B.H., Pang P.S., Ponikowski P., Voors A.A. (2019). Effects of serelaxin in patients with acute heart failure. N. Engl. J. Med..

[8] Tromp J., Bamadhaj S., Cleland J.G.F., Angermann C.E., Dahlstrom U., Ouwerkerk W., Tay W.T., Dickstein K., Ertl G., Hassanein M. (2020). Post-discharge prognosis of patients admitted to hospital for heart failure by world region, and national level of income and income disparity (REPORT-HF): A cohort study. Lancet Glob. Health.

[9] Shah K.S., Xu H., Matsouaka R.A., Bhatt D.L., Heidenreich P.A., Hernandez A.F., Devore A.D., Yancy C.W., Fonarow G.C. (2017). Heart Failure With Preserved, Borderline, and Reduced Ejection Fraction 5-Year Outcomes. J. Am. Coll. Cardiol..

[10] Steinberg B.A., Zhao X., Heidenreich P.A., Peterson E.D., Bhatt D.L., Cannon C.P., Hernandez A.F., Fonarow G.C. (2012). Trends in patients hospitalized with heart failure and preserved left ventricular ejection fraction: Prevalence, therapies, and outcomes. Circulation.

[11] Yancy C.W., Jessup M., Bozkurt B., Butler J., Casey D.E., Colvin M.M., Drazner M.H., Filippatos G.S., Fonarow G.C., Givertz M.M. (2017). 2017 ACC/AHA/HFSA focused update of the 2013 ACCF/AHA guideline for the management of heart failure: A report of the american college of cardiology/American heart association task force on clinical practice guidelines and the heart failure society of amer. Circulation.

[12] Gheorghiade M., De Luca L., Fonarow G.C., Filippatos G., Metra M., Francis G.S. (2005). Pathophysiologic targets in the early phase of acute heart failure syndromes. Am. J. Cardiol..

[13] Gheorghiade M., Zannad F., Sopko G., Klein L., Piña I.L., Konstam M.A., Massie B.M., Roland E., Targum S., Collins S.P. (2005). Acute heart failure syndromes: Current state and framework for future research. Circulation.

[14] Lang R.M., Badano L.P., Victor M.A., Afilalo J., Armstrong A., Ernande L., Flachskampf F.A., Foster E., Goldstein S.A., Kuznetsova T. (2015). Recommendations for cardiac chamber quantification by echocardiography in adults: An update from the American Society of Echocardiography and the European Association of Cardiovascular Imaging. J. Am. Soc. Echocardiogr..

[15] Nagueh S.F., Smiseth O.A., Appleton C.P., Byrd B.F., Dokainish H., Edvardsen T., Flachskampf F.A., Gillebert T.C., Klein A.L., Lancellotti P. (2016). Recommendations for the Evaluation of Left Ventricular Diastolic Function by Echocardiography: An Update from the American Society of Echocardiography and the European Association of Cardiovascular Imaging. J. Am. Soc. Echocardiogr..

[16] Chetrit M., Cremer P.C., Klein A.L. (2020). Imaging of diastolic dysfunction in community-based epidemiological studies and randomized controlled trials of HFpEF. JACC Cardiovasc. Imaging.

[17] Mckee P.A., Castelli W.P., Mcnamara P.M., Kannel W.B. (1971). The natural history of congestive heart failure: The framingham study. N. Engl. J. Med..

[18] Pieske B., Butler J., Filippatos G., Lam C., Maggioni A.P., Ponikowski P., Shah S., Solomon S., Kraigher-Krainer E., Samano E.T. (2014). Rationale and design of the SOluble guanylate Cyclase stimulatoR in heArT failurE Studies (SOCRATES). Eur. J. Heart Fail..

[19] Lam C.S.P., Rienstra M., Tay W.T., Liu L.C.Y., Hummel Y.M., Van der Meer P., De Boer R.A., Van Gelder I.C., Van Veldhuisen D.J., Voors A.A. (2017). Atrial fibrillation in heart failure with preserved ejection fraction: Association with exercise capacity, left ventricular filling pressures, natriuretic peptides, and left atrial volume. JACC Heart Fail..

[20] Pieske B., Tschö Pe C., De Boer R.A., Fraser A.G., Anker S.D., Donal E., Edelmann F., Fu M., Guazzi M., Lam C.S.P. (2019). How to diagnose heart failure with preserved ejection fraction: The HFA-PEFF diagnostic algorithm: A consensus recommendation from the Heart Failure Association (HFA) of the European Society of Cardiology (ESC) Heart failure/cardiomyopathy. Eur. Heart J..

[21] Sambataro G., Giuffrè M., Sambataro D., Palermo A., Vignigni G., Cesareo R., Crimi N., Torrisi S.E., Vancheri C., Malatino L. (2020). The Model for Early COVID-19 Recognition (MECOR) score: A proof-of-concept for a simple and low-cost tool to recognize a possible viral etiology in community-acquired pneumonia patients during COVID-19 outbreak. Diagnostics.

[22] Chioncel O., Vinereanu D., Datcu M., Ionescu D.D., Capalneanu R., Brukner I., Dorobantu M., Ambrosy A., MacArie C., Gheorghiade M. (2011). The Romanian Acute Heart Failure Syndromes (RO-AHFS) registry. Am. Heart J..

[23] Chioncel O., Tatu-Chițoiu G., Christodorescu R., Coman I.M., Deleanu D., Vinereanu D., Macarie C., Crespo M., Laroche C., Fereirra T. (2015). Characteristics of patients with heart failure from Romania enrolled in—ESC-HF Long-Term (ESC-HF-LT) Registry. Rom. J. Cardiol..

[24] Gupta K., Kalra R., Rajapreyar I., Joly J.M., Pate M., Cribbs M.G., Ather S., Prabhu S.D., Bajaj N.S. (2020). Anemia, Mortality, and Hospitalizations in Heart Failure With a Preserved Ejection Fraction (from the TOPCAT Trial). Am. J. Cardiol..

[25] Angraal S., Mortazavi B.J., Gupta A., Khera R., Ahmad T., Desai N.R., Jacoby D.L., Masoudi F.A., Spertus J.A., Krumholz H.M. (2020). Machine Learning Prediction of Mortality and Hospitalization in Heart Failure With Preserved Ejection Fraction. JACC Heart Fail..

[26] Omote K., Nagai T., Iwano H., Tsujinaga S., Kamiya K., Aikawa T., Konishi T., Sato T., Kato Y., Komoriyama H. (2020). Left ventricular outflow tract velocity time integral in hospitalized heart failure with preserved ejection fraction. ESC Heart Fail..

[27] Przewlocka-Kosmala M., Marwick T.H., Yang H., Wright L., Negishi K., Kosmala W. (2020). Association of Reduced Apical Untwisting With Incident HF in Asymptomatic Patients With HF Risk Factors. JACC Cardiovasc. Imaging.

[28] Voors A.A., Ouwerkerk W., Zannad F., Van Veldhuisen D.J., Samani N.J., Ponikowski P., Ng L.L., Metra M., Ter Maaten J.M., Lang C.C. (2017). Development and validation of multivariable models to predict mortality and hospitalization in patients with heart failure. Eur. J. Heart Fail..

[29] Pugliese N.R., Biase D., Gargani L., Mazzola M., Conte L., Fabiani I., Natali A., Dini F.L., Frumento P., Rosada J. (2021). Predicting the transition to and progression of heart failure with preserved ejection fraction: A weighted risk score using bio-humoural, cardiopulmonary, and echocardiographic stress testing. Eur. J. Prev. Cardiol..

[30] Kobayashi M., Gargani L., Palazzuoli A., Ambrosio G., Bayés-Genis A., Lupon J., Pellicori P., Pugliese N.R., Reddy Y.N.V., Ruocco G. (2020). Association between right-sided cardiac function and ultrasound-based pulmonary congestion on acutely decompensated heart failure: Findings from a pooled analysis of four cohort studies. Clin. Res. Cardiol..

